# CD151 in Respiratory Diseases

**DOI:** 10.3389/fcell.2020.00064

**Published:** 2020-02-07

**Authors:** Amanda H. Wong, Thai Tran

**Affiliations:** Department of Physiology, Yong Loo Lin School of Medicine, National University of Singapore, Singapore, Singapore

**Keywords:** CD151, lung cancer, asthma, influenza, idiopathic pulmonary fibrosis, biomarker, targeted therapy, tetraspanin

## Abstract

The tetraspanin, Cluster of Differentiation 151 (CD151), is ubiquitously expressed in adult tissue, especially in the lungs where it has been implicated in lung cancer, asthma, influenza, and idiopathic pulmonary fibrosis (IPF). CD151 interacts with laminin-binding integrins and growth factor receptors, and is reported in cancer-promoting processes such as tumor initiation, metastasis, and angiogenesis. In asthma, CD151 was shown to promote airways hyperresponsiveness through calcium signaling whereas in influenza, CD151 was shown to be a novel host factor for nuclear viral export signaling. Furthermore, CD151 was shown to be associated with increased disease severity and poorer survival outcome in asthma and lung cancer, respectively. In this review, we provide an update on the current understanding of CD151 with regards to its contribution to lung pathophysiology. We also summarize factors that have been shown to regulate CD151 expression and identify key areas that need to be taken into consideration for its utility as a screening or prognostic tool in disease management and/or as a therapeutic target for the treatment of lung diseases.

## Introduction

Respiratory diseases account for significant illness and premature mortality around the world. The global impact of respiratory diseases is difficult to quantify due to discrepancies or insufficient data across regions, however, the socioeconomic burden of these conditions cannot be ignored. More than 9.5 million deaths globally were attributed to respiratory diseases – the most common of which were asthma, lower respiratory infections, chronic obstructive pulmonary disease, and lung cancer – and the total cost of respiratory diseases in the European Union alone was estimated to total over €380 billion annually (Gibson et al., 2013; Ferkol and Schraufnagel, 2014). Whilst risk factors such as air pollution, tobacco smoke exposure, occupational agents, and genetics (Gibson et al., 2013) have been identified, respiratory diseases are not curable in some cases and current treatment options are suboptimal for the majority of patients with chronic respiratory diseases. Hence, the development of novel therapies is required to alter the progression of disease severity and/or prevent disease onset.

**FIGURE 1:**
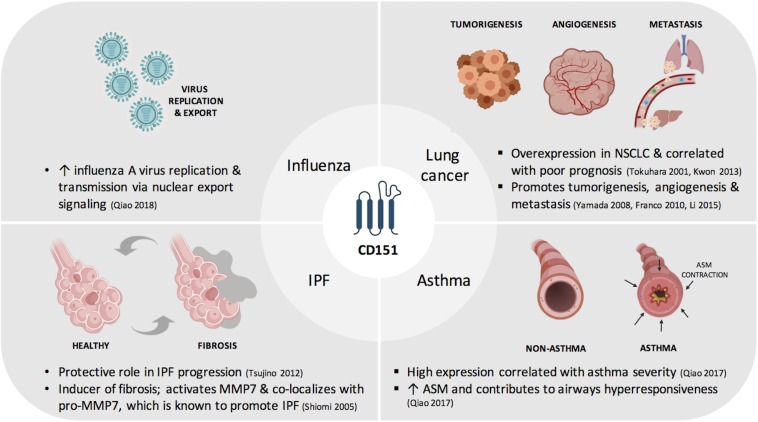
Contribution of CD151to respiratory diseases. ASM, airway smooth muscle; IPF, idiopathic pulmonary fibrosis; MMP7, matrix metalloproteinase-7; NSCLC, non-small cell lung cancer.

In this review, we provide an update on the current understanding of CD151 with regards to its contribution to lung physiology and pathophysiology (Figure 1). We also summarize factors that have been shown to regulate CD151 expression and identify key areas that need to be taken into consideration for its utility in disease management as a screening or prognostic tool and as targeted or adjuvant therapy.

## CD151

Cluster of Differentiation 151 was first identified as a platelet surface glycoprotein, gp27, where it was found to induce platelet aggregation and mediator release (Ashman et al., 1991). Following successful cloning of the cDNA encoding gp27, it was shown to belong to the transmembrane 4 superfamily, tetraspans, and renamed Platelet-Endothelial cell Tetra-span Antigen-3 (PETA-3) (Fitter et al., 1995). The following year, another team independently identified the same protein but named it protein SFA-1 as it was induced by a T-cell leukemia cell line, SF-HT. The protein was subsequently reassigned as Cluster of Differentiation 151 (CD151) (Ashman, 2002). Whilst early reports identified CD151 as a surface protein, CD151 is also expressed intracellularly (Hwang et al., 2019), including cytoplasmic vesicles, endothelial cell junctions and perinuclear regions (Liu et al., 2007; Qiao et al., 2017).

Like other tetraspanin family members, CD151 forms lateral associations with multiple partner proteins within the tetraspanin-enriched microdomain (TEM). The most prominent partners of CD151 are laminin-binding integrins (Liu et al., 2007), whilst adhesion molecules, growth factor receptors, metalloproteinases, and intracellular signaling molecules have also been shown to be associated with CD151 or be localized within TEMs (Hemler, 2003; Stipp et al., 2003). In addition to membrane clustering and organization, they have been shown to modulate, stabilize or prevent the activities of their associate molecules to influence cell activation, proliferation, migration, adhesion, and signaling.

Cluster of Differentiation 151 is widely expressed in multiple cell types of normal human tissue (Sincock et al., 1997). Immunohistochemical staining showed that CD151 was found to be strongly positive in the lungs, specifically in airway smooth muscle (Qiao et al., 2017), epithelial layers, endothelial cells and blood vessels (Sincock et al., 1997). Given its abundance in lung tissue, the role of CD151 in normal and pathological processes in airways is especially relevant.

### CD151 in Development and Maintenance of Lung Structure

The role of CD151 in normal lung physiology is not fully explored but inferences about its critical role can be made from clinical cases of CD151 gene mutation and from studies in mice where CD151 is deleted. With regards to clinical cases of CD151 gene mutation, there have been six reported case studies of patients with CD151 gene mutations resulting in truncated or altered forms of the protein. Nephropathy was reported in four of the cases as well as epidermolysis bullosa and sensorineural deafness in two cases (Karamatic Crew et al., 2004; Vahidnezhad et al., 2018). The remaining two cases, with a single base substitution, exhibited no significant clinical outcomes (Karamatic Crew et al., 2008). Differences in lung function or morphology were not reported in these patients – whether genetic mutations of CD151 had no effect on lung physiology and therefore it was not reported, or whether the investigators did not look at other organ phenotypes is not known. In CD151-null mice, several studies have recapitulated the significant role of CD151 in kidney, skin and inner ear function (Wright et al., 2004; Sachs et al., 2006). Interestingly, Tsujino et al. (2012) reported that CD151 is essential for the normal function of alveolar epithelial cells (APCs) as the deletion of CD151 in APCs resulted in the loss of epithelial integrity (loss of epithelial adhesion to the basement membrane). In addition, there were increases in collagen deposition, collagen-1 expression and hydroxyproline content in the lungs of the CD151-null mice and lung compliance was also reduced in these mice as compared to wildtype control mice. The authors concluded that CD151 functions to protect against pulmonary fibrosis by maintaining epithelial integrity.

Besides these reports, findings from other groups may be informative in understanding the physiological role of CD151. Treatment with anti-CD151 antibody resulted in the loss of epithelial integrity in skin epithelial cells (Shigeta et al., 2003) whilst CD151 knockdown was shown to disrupt integrin-mediated adhesion to the basement membrane (Yamada et al., 2008). Both these processes are crucial for the development and maintenance of lung structure. Given the strong interaction between CD151 and its laminin-binding partners, defects in bronchi morphogenesis and lung deficiencies observed with the deletion of integrin α3β1 (Kreidberg et al., 1996) and laminin-α5 (Nguyen et al., 2002), respectively, cannot be overlooked and further studies have to be conducted to conclusively determine whether the physiological conditions observed are a direct consequence of CD151 dysregulation or a secondary effect of CD151 due to disruption of its laminin-binding partners.

### Tumor Promoter in Lung Cancer

Lung cancer has a high degree of molecular heterogeneity thus giving rise to diverse pathological features. Cases are classified according to respective histological characteristics leading to the distinction between small and non-small cell lung cancer (NSCLC) (Herbst et al., 2008). The onslaught of molecular targets has driven a shift in therapeutic management from conventional cytotoxic drugs to targeted molecular targeted therapy (Hirsch et al., 2017; Herbst et al., 2018). The pathogenesis of lung cancer and its management has been covered extensively by others in the preceding reviews. CD151 was the first tetraspanin to be identified as a tumor promoter (Testa et al., 1999). The authors found that the monoclonal antibody raised against CD151 was capable of inhibiting both spontaneous and experimental metastasis in the chick embryo chorioallantoic membrane model. Since then, CD151 overexpression has been implicated as a negative prognostic indicator in numerous solid malignancies (Ang et al., 2004; Chien et al., 2008; Ke et al., 2009; Suzuki et al., 2011; Kwon et al., 2012; Lee et al., 2012; Kang et al., 2013; Romanska et al., 2013; Fisher et al., 2016; Medrano et al., 2017).

In NSCLC, several studies have shown that CD151 overexpression, measured by gene expression and immunohistochemical analysis, is associated with poorer prognosis in terms of survival rate (Tokuhara et al., 2001) or overall and disease-free survival (Kwon et al., 2013). Exosome protein profiling comparing a cohort of lung cancer patients with non-cancer patients revealed that CD151 was one of the markers found to be upregulated in lung cancer (Sandfeld-Paulsen et al., 2016). This study showed that whilst CD151 expression was detected on structural or immune cells, these cells also secreted CD151-containing exosomes in circulation to act as components for crosstalk between cancer initiating cells and their environment (Yue et al., 2015). This highlights their key role in cancer and as a mediator of cell-cell communication.

Use of gene deletion technology and/or use of monoclonal antibodies directed at CD151 have been employed to explore the functional significance of CD151. In lung cancer cells, CD151 knockdown resulted in impairment of several cancer-promoting processes including cell survival, migration, invasion, and matrix adhesion. These changes were mainly due to CD151 interaction with partner or associated proteins such as integrin α3β1 (Yamada et al., 2008), matrix metalloproteinase-9, vascular endothelial growth factor (Li et al., 2015) and hepatocyte growth factor (Franco et al., 2010), and subsequently downstream signaling of these proteins. In spontaneous lung metastasis models, CD151-null mice exhibited a marked delay in tumor initiation and a decrease in number of metastastic lesions (Deng et al., 2012; Copeland et al., 2013). Similarly, when tumor cells were injected at a separate primary site, CD151-null mice also showed reduced lung metastasis and tumor cell residence (Takeda et al., 2007, 2011; Sadej et al., 2010).

### Hyperresponsiveness Mediator in Asthma

Asthma is a chronic airway disease characterized by three main features, airways hyperresponsiveness, airway wall remodeling and airway inflammation (Barnes, 1996; Kudo et al., 2013). Current asthma treatment is mainly focused on long-term clinical control of asthma and rapid-acting symptom relief. However, these drugs are suboptimal in controlling disease, for example in the case of the widely prescribed inhaled corticosteroids which are limited in its clinical efficacy due to varying responsiveness in the population and increased risk of adverse effects when administered at higher doses (Bateman et al., 2008; Reddel et al., 2015). Therefore, a better understanding of underlying mechanisms of disease and therapeutic targeting of these mechanisms may contribute greatly to improved asthma management.

Expression of CD51 is clinically relevant to asthma as immunohistochemical analysis of human bronchial biopsy specimens for CD151 expression in airway smooth muscle bundles were highest in mild or moderate asthma subjects compared with non-asthmatic subjects (Qiao et al., 2017). In the same study, CD151 was implicated to play a role in airways hyperresponsiveness – loss- and gain-of-function studies showed that CD151 enhances G protein-coupled receptor (GPCR)-induced calcium and protein kinase C signaling, which are key signaling pathways implicated in airway smooth muscle cell contraction. In support, CD151-null mice did not exhibit increased airway reactivity to contractile agonists and were shown to have increased lung compliance compared to the wild-type group (Qiao et al., 2017). This study is significant as it provides a better understanding of the mechanism for airways hyperresponsiveness which may help in the design of more efficacious and emerging therapies for asthma. What remains to be determined is whether high CD151 expression is a result of asthma or whether elevated expression of CD151 results in asthma. Furthermore, the impact of CD151 on the remaining key features of asthma, airway wall remodeling, and airway inflammation, is yet to be established.

### Host Factor in Influenza Viral Export

An estimated 5% of adults develop influenza symptoms annually with severe cases leading to conditions such as bronchitis and pneumonia. These seasonal epidemics are largely due to influenza viruses A and B. Influenza viruses are segmented, negative-sense RNA viruses within a lipid envelope containing the surface glycoproteins hemagglutinin and neuraminidase – involved in viral entry and progeny release, respectively (Nicholson et al., 2003; Shim et al., 2017). Tetraspanins are reported to play critical roles in multiple virus life cycle processes such as virus entry, endocytosis, intracellular trafficking, assembly and budding (van Spriel and Figdor, 2010). CD151 was previously implicated in several viral infections including papillomavirus, cytomegalovirus and human immunodeficiency virus (Thali, 2011; Scheffer et al., 2014; Hochdorfer et al., 2016). CD151 plays a critical role in influenza A virus signaling (IAV) (Qiao et al., 2018). The knockdown of CD151 expression in patient-derived nasal epithelial cells resulted in a significant reduction in IAV progeny viral load. In addition, CD151-null mice were more resistant and tolerant to IAV infection with higher survival rates as compared to the wild-type infected mice. With regards to respiratory infection, the anti-viral responses were attributed to CD151 mediating the export of progeny viruses through its binding to newly synthesized viral proteins (NP, M1, and NEP) and host nuclear export protein (CRM1). The novel action of CD151 on viral nuclear export signaling associated with H3N2 and H1N1 infection (Qiao et al., 2018) highlights the potential of CD151 to be developed as a novel therapy to treat influenza and other viral infections. Targeting CD151 is especially beneficial given that it targets a host mechanism thus making it less susceptible to constant changes in viral strains – due to antigenic shift and antigenic drift – that render current antiviral drugs or vaccines ineffective after each season.

### Protective or Progressive Role in Idiopathic Pulmonary Fibrosis

Idiopathic pulmonary fibrosis (IPF) is a form of interstitial pneumonia that features the chronic and progressive scarring of the lung. Initially regarded as an inflammatory disease, recent reports have highlighted the importance of AECs activation in causing repetitive injury in IPF (King et al., 2011; Richeldi et al., 2017). This triggers the recruitment of immune cells and fibroblasts in the lung microenvironment and initiates the remodeling of the extracellular matrix and lung regeneration (Martinez et al., 2017; Mora et al., 2017).

Interestingly, CD151-null mice spontaneously developed pulmonary fibrosis features observed at 30 weeks and exhibited accelerated bleomycin-induced lung injury in the pulmonary fibrosis model, suggesting a protective role of CD151 in IPF. This was attributed to the critical function of CD151 in maintaining AECs integrity, and that CD151 deletion attenuates AECs adhesion to the basement membrane, upregulates transforming growth factor-β1 (TGF-β1) signaling in AECs and promotes epithelial-mesenchymal transition changes. In support of this, CD151-negative AECs were focally observed in lung biopsies from IPF patients. However, it is unclear if the IPF phenotype observed in CD151-null mice is strain-specific as differences in renal function in tumor formation (Li et al., 2013) has been observed between FVB/N background [generated using 129SvEv ES cells (Takeda et al., 2007; Tsujino et al., 2012)] versus C57BL/6 mice [generated from 129/Ola and C57BL/6 ES cells (Wright et al., 2004)]. Importantly, whether similar phenotypes are observed in patients with CD151 mutations remains to be explored.

In contrast to the report above describing the protective role of CD151 in IPF, Fujishima et al. (2010) proposed that interactions between CD151 and MMP-7 (for which expression is elevated in IPF patients) could contribute to worsening of IPF condition. They show that CD151 facilitates MMP-7 activation by acting as a docking molecule (Shiomi et al., 2005) and was colocalized with pro-MMP-7 in sections of lung tissue from IPF patients. However, CD151 levels in these IPF patient samples were not directly measured in this study (Fujishima et al., 2010). In support, CD151 has been implicated in other fibrotic processes, such as liver fibrosis and skin wound healing (Mazzocca et al., 2002; Geary et al., 2008). Therefore, more studies are warranted to clearly define the role of CD151 in fibrosis in general and IPF more specifically.

## Regulation of CD151 Expression

### CD151 Expression Modulators

Given the importance of CD151 in respiratory diseases, it is interesting to note that little is known about regulators of CD151 expression changes. To date, there are only two studies that report that the expression of CD151 mRNA and protein abundance are modulated by therapies – anti-epileptic drugs and anti-cancer drugs, respectively (Hua et al., 2001; Hwang et al., 2019). The study conducted by Hua et al. (2001) showed that chronic administration of the anti-epileptic drugs, carbamazepine or valproate, significantly decreased CD151 transcript levels in rat frontal cortex following chronic (five-week) treatment. The functional significance of this reduction was not explored in this study and there have been no followup studies to date by this group or others. In contrast to that, a recent study by Hwang et al. (2019) showed that several anti-cancer drugs (gefitinib, lapatinib, cisplatin, oxaliplatin, camptothecin, and 5-fluorouracil) induced CD151 protein levels in A431 skin epidermoid carcinoma cells. They further showed that gefitinib upregulated CD151 protein levels in MDA-MB-231 breast and A549 lung carcinoma cell lines, respectively. In this study, the group showed that anti-cancer drug resistance may in part be attributed to CD151 upregulation, for which CD151 knockdown sensitized the cells to drug treatment. It remains to be seen whether these effects could be translated to *in vivo* conditions.

Besides drug-induced regulation, hypoxic conditions were shown to regulate CD151 expression and subsequently cell adhesion and metastasis. Hypoxia is the condition of oxygen deficiency and is a major driver of cancer-promoting processes such as angiogenesis and migration. Hypoxia is mainly mediated through hypoxia-inducible factors (HIF) (Ke and Costa, 2006). In this study, the expression of CD151 was downregulated under hypoxic conditions via the HIF-1α-dependent pathway in colorectal cells. HIF-1α dependency was further confirmed when CD151 levels were inhibited by HIF-1α induction through treatment with desferrioxamine (hypoxia-mimetic agent) or overexpression with plasmid vectors (Chien et al., 2008). It remains to be observed whether the same hypoxic conditions can reduce CD151 in a lung cancer setting.

### Mechanisms Underlying CD151 Regulation

Whilst CD151 expression modulation plays a critical role in determining disease progression, whether in promoting pathophysiology of lung cancer, asthma, and influenza or in potentially protecting against IPF, there is a distinct gap in the literature pertaining to the mechanisms underlying these changes. In the study reporting CD151 mRNA downregulation after treatment with anti-epileptic drugs, valproate and carbamazepine (Hua et al., 2001), there were no experiments conducted to explain gene expression changes or whether downstream protein expression was affected. Whereas in the anti-cancer drug-induced CD151 study (Hwang et al., 2019), the authors ruled out integrin dependence and proposed that CD151 upregulation may be due to diminished protein degradation. However, there are no reports to date to corroborate this proposal.

Interestingly, the mechanism underlying hypoxia-mediated downregulation of CD151 was determined to be at the transcriptional level (Chien et al., 2008). In addition to the decrease in CD151 protein expression under hypoxic conditions, CD151 mRNA levels were also reduced significantly in hypoxic compared to normoxic conditions. This reduction in mRNA levels was confirmed with desferrioxamine treatment. The group went on to identify a putative hypoxia-response element (HRE) in the human CD151 promoter region and intron II, suggesting a direct action on CD151 regulation by hypoxia exposure.

In addition to transcriptional regulation of CD151 expression, another area that may be explored is post-translational modifications. Several reports have previously identified six intracellular C- terminal cysteine sites on CD151 that are palmitoylated, that is, C11, C15, C79, C80, C242, and C243 (Berditchevski et al., 2002; Yang et al., 2002). Besides palmitoylation, CD151 may also undergo glycosylation at the asparagine residue, N159 (Baldwin et al., 2008). However, palmitoylation and glycosylation modifications have not exhibited differences in expression levels or staining pattern (Baldwin et al., 2008; Zevian et al., 2011). Also, the effect of drug treatment on these post-translational modifications has not been elucidated. Besides palmitoylation and glycosylation of CD151, the post-translational modification of ubiquitination should also be explored as it may be informative with regards to CD151 protein stability.

## Key Considerations for CD151 in the Management of Lung Diseases

### CD151 as a Prognostic and Diagnostic Tool

The prognostic value of CD151 was previously emphasized in low-grade prostate cancer, in which CD151 expression could predict the clinical outcome of patients more accurately than the traditional histological grading method (Ang et al., 2004). Given the strong association between CD151 and lung cancer (Tokuhara et al., 2001; Kwon et al., 2013), especially adenocarcinoma which is the most common NSCLC subtype, CD151 expression has proven to be especially informative on patient prognosis. Furthermore, the prognostic utility of CD151 expression may be extended to other diseases such as asthma, in which the expression of CD151 was associated with disease severity (Qiao et al., 2017).

Besides immunohistochemical analysis, an emerging technology in determining protein expression is exosome protein profiling. Tetraspanins, including CD151, are shown to be highly enriched in exosomes (Merino et al., 2014) which may be derived from plasma or urine samples (Jakobsen et al., 2015) making this method far less invasive than traditional biopsy techniques. The potential of CD151 expression detection in exosomes as a screening tool has been explored where a high level of accuracy (72%) in detecting cancer in adenocarcinoma patients was observed (Sandfeld-Paulsen et al., 2016). Effective screening methods are especially vital in NSCLC for which 60% of patients are only diagnosed at an advanced stage (Reck et al., 2013). Whilst this method requires further validation, it may be a promising avenue to complement or improve current diagnostic processes.

### Targeted CD151 Therapy

Given the role of CD151 in a milieu of processes that contribute to lung disease pathophysiology, targeting CD151 in a clinical setting is justifiable. The most commonly reported method of targeting CD151 is with anti-CD151 monoclonal antibodies. Treatment with these antibodies has been shown to impair cellular processes at multiple cancer stages. Tumor growth potential, neoangiogenesis and metastasis following injection with a hepatocellular carcinoma cell line was also inhibited in mice that were treated with mAb 9B, which was shown to specifically disrupt the interaction between CD151 with integrin α6β1 (Ke et al., 2011). Two other studies recapitulated the anti-metastasis action in chick embryo models using the monoclonal antibodies, mAb 50-6 (Testa et al., 1999) and mAb 1A5 (Zijlstra et al., 2008), which were shown to reduce both spontaneous and experimental metastasis. Similarly, the mAb SFA1.2B4 was shown to impair pulmonary metastasis in mice injected with either colon cancer or fibrosarcoma cell lines (Kohno et al., 2002). Whether or not these monoclonal antibodies disrupt disease progression in respiratory diseases remain to be explored, although its anti-cancer properties for the treatment of lung cancer are promising.

Besides the use of monoclonal antibodies, CD151 gene deletion has been instrumental in improving markers of disease outcomes in asthma, influenza, and tumor progression. As described above, CD151 knockdown, through siRNA technology, markedly reduced airway smooth muscle contraction whilst airway hyperresponsive processes decreased significantly in allergen-induced CD151-null mice. Following influenza A virus infection, CD151-null mice exhibited better survival and reduced viral titer, which was attributed to the role of CD151 in nuclear export of viral proteins confirmed using CD151 knockdown experiments. In the context of cancer, both experimental lung metastasis and tumor cell residence were reduced in CD151-null mice. Furthermore, pathologic angiogenesis was impaired in these CD151-null mice despite showing no vascular defects under normal developmental conditions. The impact of CD151 was also reported in breast cancer in which mammary tumor initiation, tumor growth, survival, and metastasis were impaired in CD151-null mice (Yang et al., 2008; Deng et al., 2012).

Despite the promising results with monoclonal antibodies and gene deletion, there are several obstacles that restrict the development of these therapeutic strategies. The ubiquitous expression of CD151 in normal human tissue (Sincock et al., 1997) and its role under physiological conditions (as described above) warrant further studies to determine whether diminishing CD151 expression for therapeutic benefit in the lungs may lead to unwanted, adverse effects to other healthy cells/organs. In this regard, advancements in targeted drug delivery would be extremely beneficial to avoid off-target effects. Developments in the field of nanotechnology and the advent of nanoparticles or nanocarriers in biomedical research provide another avenue for exploiting the importance of CD151 in lung diseases. Several reviews have summarized the clinical significance of nanomedicine in which binding molecules (such as peptides, aptamers, and antibodies) are utilized in cell-specific or tissue-localized delivery of drugs, compounds or genetic material (Poelstra et al., 2013; Rosenblum et al., 2018). For instance, using the nanoparticle-based system, Deshmukh et al. (2018) were able to deliver the small molecule EGFR-specific inhibitor, erlotinib, specifically to myofibroblasts by means of targeting a transmembrane receptor, platelet-derived growth factor receptor-beta, which is uniquely expressed on myofibroblasts in the liver. This specific delivery to hepatic myofibroblasts was more effective and well-tolerated compared to systemic administration of the drug alone (Deshmukh et al., 2018). No studies to date have reported the use of CD151-specific binding molecules as a targeting mechanism for drug delivery, but its potential could be explored given the importance of CD151 overexpression in various lung diseases and other human malignancies. Delivery of genetic material to inhibit the expression of CD151, such as in the case of short hairpin RNA or guide RNA for CRISPR/Cas9-based genome editing, is another approach that may be investigated. Also, advancements could be made to improve not only uptake but enhanced retention of drugs (Matsumura and Maeda, 1986; Klibanov et al., 1990; Blume et al., 1993; Blanco et al., 2015), as well as route of drug administration (local delivery via inhalation versus systemic delivery) (Yan et al., 2016; Cryer and Thorley, 2019; Mukherjee et al., 2019). In addition to that, the magnitude of CD151 reduction required in cases of overexpression will also need to be assessed, specifically whether CD151 expression levels can be ablated completely or must be brought back to a baseline expression to ensure physiological functions are not disrupted. Finally, drug-induced upregulation of CD151 must be taken into account given the importance of expression changes in disease progression and severity.

Cluster of Differentiation 151 may also play an important role as an adjuvant therapy alongside currently available therapeutics. Given the close association of CD151 with oncogenic drivers including growth factors, such as EGFR (Yang et al., 2008; Deng et al., 2012), HER2 (Yang et al., 2010; Deng et al., 2012; Romanska et al., 2015), and HGFR (c-Met) (Klosek et al., 2005; Franco et al., 2010; Ha et al., 2014), CD151 was shown to impair growth factor receptor-dependent processes such as tumor onset, cell growth, spreading and motility. CD151 ablation in these studies was sufficient to arrest or impair oncogenic responses. Furthermore, in the case of EGFR and HER2-specific inhibitors, drug sensitivity was restored when CD151 expression was abrogated (Hwang et al., 2019). Restoration of drug sensitivity with CD151 knockdown was shown to not be restricted to targeted therapy but also observed in chemotherapeutic drugs oxaliplatin, cisplatin, and 5-fluorouracil, via apoptotic induction.

## Conclusion

Cluster of Differentiation 151 is involved in numerous physiological and pathophysiological processes. Therefore, it has been implicated in several diseases, including respiratory diseases such as lung cancer, asthma, influenza and IPF (Figure 1). The majority of studies link CD151 to disease onset, severity or progression. The mechanisms underlying CD151 upregulation or downregulation remain to be fully understood. Nonetheless, the clinical significance of CD151 expression shows that CD151 has great potential to be developed as a diagnostic biomarker which may be helpful for early detection or screening, or as a prognostic biomarker given its relevance in predicting lung cancer or asthma severity. Furthermore, CD151 monoclonal antibodies, gene deletion, and nanotechnology studies support the notion for its development as a novel targeted or adjuvant therapy in the treatment of respiratory diseases.

## Author Contributions

AW and TT wrote and edited the manuscript.

## Conflict of Interest

The authors declare that the research was conducted in the absence of any commercial or financial relationships that could be construed as a potential conflict of interest.
